# Symptom Burden of Children with Cancer and Parental Quality of Life: The Mediating Role of Parental Stress

**DOI:** 10.3390/ijerph19169840

**Published:** 2022-08-10

**Authors:** Winsome Lam, Su-Fang Li, Yan-Zhi Yi, Ka Yan Ho, Katherine K. W. Lam, Doris Y. P. Leung, Kitty Y. Y. Chan, Jacqueline M. C. Ho, Stephen C. W. Chan, Hai-Xia Wang, Li Zhou, Yan Yin, Frances K. Y. Wong

**Affiliations:** 1 School of Nursing, The Hong Kong Polytechnic University, Hong Kong, China; kyeva.ho@polyu.edu.hk (K.Y.H.); kw-katherine.lam@polyu.edu.hk (K.K.W.L.); doris.yp.leung@polyu.edu.hk (D.Y.P.L.); mc-jacq.ho@polyu.edu.hk (J.M.C.H.); kitty.yy.chan@polyu.edu.hk (K.Y.Y.C.); frances.wong@polyu.edu.hk (F.K.Y.W.); 2 Nursing Department, Shenzhen Children’s Hospital, Shenzhen 518038, China; etyyhlb6013@126.com (S.-F.L.); wangshiqi2017@126.com (H.-X.W.); 3 Pediatric Department, University of Hong Kong-Shenzhen Hospital, Shenzhen 518000, China; nkdyyhlb@163.com (Y.-Z.Y.); zhoul6@hku-szh.org (L.Z.); 4 Caritas Medical Centre, Hong Kong, China; stephencwchan@ha.org.hk; 5 Department of Pediatric Hematoncology, Shenzhen Children’s Hospital, Shenzhen 518038, China; yinyan0915@sina.com

**Keywords:** pediatric cancer, symptom burden, parental stress, parental quality of life

## Abstract

Background: The aim of this study was to investigate the association between children’s reported symptom burden and their parents’ quality of life, and whether parents’ perceived stress mediates this relationship. Method: this was a cross-sectional quantitative research study. Convenience sampling was used to recruit 80 pairs of parents and their children with cancer. Advanced statistical methods were used to analyse the mediating effects of parental stress between children’s symptom burden and parents’ quality of life. Results: The results showed that parental stress was the mediator in the relationship between children’s reported symptom burden and their parents’ quality of life. Conclusions: Symptom burden was prevalent in Chinese children with cancer living in the community. Children’s symptom burden is an important factor in predicting parental stress level, which simultaneously and directly lower parents’ quality of life. The evidence in this study enlarges the knowledge base about the mediating effect of parental stress on the association between the symptom burden of children with cancer and their parents’ quality of life. This evidence is crucial in paving the way for the development of interventions that improve the parental quality of life through stress-reduction programs.

## 1. Introduction

Cancer remains the leading cause of mortality among children [1]. Resulting from the advancement in medical technology, the survival rate of most types of childhood cancer has now improved to approximately 80% [2]. Despite advances in health care, the majority of these children living with cancer in their community experience one or more physical symptoms (i.e., low appetite, fatigue, pain etc.) related to their cancer or treatment late effects along with psychological distress such as sadness, worry etc. [3,4]. Suffering from this symptom burden negatively impacts children’s physical and psychosocial condition and lowers their quality of life in terms of physical, psychosocial and school function [5]. Nevertheless, the symptom burden is not only a disruptive presence in children’s lives, but also in their parents’ lives [6]. The diagnosis and disease treatment, along with caregiving activities for the children with cancer, induce different emotional distress such as depressed moods, and worries in these parents [7,8]. Parents, as primary caregivers, always spend a lot of time with their sick children in the activities of daily living and managing their children’s symptoms at home. All these might result in high levels of stress to parents. If parents’ stress is not managed well, it may have a deleterious impact on both parents’ and children’s quality of life [7,9]. This is a vicious cycle further hindering parents’ abilities and confidences in providing quality care to their unwell children living at home.

This postulation is in-line with the assumption of reciprocal determinism based on Attachment Theory, namely that there is a mutual impact between parents and their children [10]. Particularly, the severity of the symptom burden reported by children may increase the level of parents’ stress [11,12,13,14]. Although parental stress appears to be a mediator between the relationship between children’s symptom burden and parents’ quality of life, this postulation remains a hypothetic discussion based on Attachment Theory. In fact, there is a paucity of studies to investigate the associations among children’s symptom burden, parents’ stress level, and parental quality of life. If parents could well manage their own stress, they might be more successful in caring for their children’s medical needs [15]. Therefore, this research study aimed at providing a clear understanding of these associations and further look into the mediating effect of parental stress on the relationships between children’s symptom burden and parents’ quality of life. Investigating the mediation effect of these variables is of great importance in providing explanations for the health outcomes of parents [16]. It would assist paediatric nurses in detecting parents who are vulnerable to suffering from stress and poor quality of life and provide prompt nursing care for parents in need. Such knowledge promises to improve the ability of the nursing profession and pave the path for intervention development to support parents in managing symptoms for their children, as well as parents’ own stress from the chronic sequelae of cancer treatment [17].

## 2. Methods

### 2.1. Study Design

This was a quantitative cross-sectional study design, using a questionnaire with closed-ended questions to collect the data.

### 2.2. Sample

A convenience sampling approach was adopted to recruit 80 parents and their children living with cancer in this study. All the children and parents were Chinese and living at home in the community. The inclusion criteria for the children were (1) having any type of paediatric cancer; (2) being aged between 10 and 18 years old; (3) able to communicate in and read Chinese. The inclusion criteria for the eligible parent were (1) one of the parents of the child with cancer and (2) able to communicate in and read Chinese.

The exclusion criteria were (1) the child being in survivorship; (2) the child being newly diagnosed with cancer within the previous 12 months; (3) the child being admitted to hospital in the previous seven days; (4) the parent or child being reported as having a mental disorder; and (5) the child and parent being under end-of-life care.

### 2.3. Data Collection

The eligible parents and children were screened and referred to the research team by paediatricians from a participating hospital in Hong Kong, and two participating hospitals from Shenzhen, China during the out-patient consultations from December 2019 to January 2021. A total of 130 parents and their children were invited to join the research study. 80 parents and their children accepted the invitation and completed the surveys within the project period. The response rate was 61.5%. The research team then approached the parents and their children and gave them an explanation of the purpose of this study. After obtaining the written consent from parents and children, they were interviewed independently in an interview room provided by the hospital clinics. Questionnaires using a self-administered approach were adopted for data collection. An explanation on how to complete the questionnaire was given to parents and children before the interviews started.

### 2.4. Measures

Parents’ age, marital status, education level, insurance coverage for the child, and financial status were gathered as the demographic data. In order to provide a context for the child and his/her parent, child’s age, diagnosis, disease status, total number of hospital admissions in the last year, and the total number of hospitalized days in all admissions in the last year were also obtained.

#### 2.4.1. Memorial Symptom Assessment Scale (MSAS 10-18) (Chinese Version)

The 30-item MSAS, consisting of three subscales: a physical subscale, a psychological subscale, and a global distress index, was adapted to measure the occurrence, frequency, intensity and distress of child-reported symptoms in the past seven days [4,18]. Dichotomous questions were used to measure the occurrence of symptoms. Frequency and intensity of a symptom were rated on the items with a four-point Likert scale from 1 (almost never) to 4 (always). The items for measuring distress were ranked on a five-point Likert scale from 1 (not at all) to 5 (very). The total score calculation was based on the responses from the 30 items, with a range of 0—4 per item and a greater score indicating a greater level of children’s symptom burden. The Cronbach’s alpha of this assessment tool in current samples was 0.869. The overall reliability was satisfactory, with a Pearson’s correlation coefficient of 0.95 and a Cronbach’s alpha value of 0.87.

#### 2.4.2. Perceived Stress Scale (PSS-10) (Chinese Version)

This is a 10-item version of the PSS (PSS-10), used to measure the degree to which situations in the parents’ lives were evaluated as stressful [19]. The Cronbach’s alpha value of this PPS-10 was 0.603 in the existing samples. Parents rated each item on a five-point Likert scale, where 1 indicated low (not at all) and 5 indicated high (extremely). The total score calculation was based on the sum of the 10 items, with a range from 0–40. A higher score indicated greater parental stress.

#### 2.4.3. WHOQOL-SRPB-BREF (Chinese Version)

The WHOQOL-SRPB instruments consisted of 34 questions, covering quality of life aspects related to spirituality, religiousness and personal beliefs (SRPB). Individual items were rated by the parent on a five-point Likert scale, where 1 indicated low (negative perception) and 5 indicated high (positive perception). The coefficient alpha reliability was 0.8 [20]. The overall score of the WHOQOL-SRPB-BREF ranged from 0 to 170, with a higher score indicating better parental quality of life. The Cronbach’s alpha of the WHOQOL-SRPB- BREF was 0.938 in the samples in the present study.

### 2.5. Ethical Considerations

Ethical approvals were obtained from the Institute Review Board of the University and the three participating out-patient clinics before data collection started. Written consent was obtained from both children and parents, and they were provided with a detailed explanation of the study aim, procedure and ethical concerns before the interviews commenced. The names of the children, the parents, and the clinics were kept anonymous in any publication. The parents and children were informed that participating in this research was voluntary and they could withdraw at any time. No parent or child reported any distress condition during the interviews.

## 3. Statistical Analysis

The sociodemographic characteristics of the participants were examined by computing frequencies, percentages, means, and standard deviations whenever appropriate. The z-statistic tests based on skewness and kurtosis were used to check for the normality of the data on the continuous variables [21]. For a medium sample size ranging from 50 to 300, an absolute value of the z-statistic > 3.29 indicates non-normality. Sociodemographic differences in children’s symptom burden, parents’ perceived stress, and parents’ quality of life were examined using parametric tests (independent *t*-test, ANOVA, and Pearson’s correlation) for normally distributed variables and non-parametric tests (the Mann-Whitney U test, the Kruskal-Wallis H test, and Spearman’s correlation) for non-normally distributed variables. The interrelationships among children’s symptom burden, parents’ perceived stress, and parents’ quality of life were examined using either Pearson’s or Spearman’s correlation. We also conducted mediation analysis to examine whether parents’ perceived stress mediated the relationship between children’s symptom burden and parents’ quality of life. The PROCESS macro version 3.4 with the regression bootstrapping method [22] was performed to determine the mediating effect of parents’ perceived stress and the direct and indirect effects of children’s symptom burden on parents’ perceived stress and quality of life. Demographic variables that were found to be significantly associated with the study variables were also included in the mediation analysis to adjust for their effects. In the mediation analysis, a 95% confidence interval (CI) was computed based on 5000 bootstrap samples generated by the PROCESS. All analyses were performed using SPSS version 26, and the significance level was set at 5%.

## 4. Results

### Sociodemographic Characteristics

A total of 80 children with cancer and their parents were included in this study. Details of the participants’ demographic characteristics are reported elsewhere [23], but they can briefly be summarized as follows. Of the 80 children, 51.2% were diagnosed with haemopoietic malignancies and 48.7% were diagnosed with tumours. 45.0% were female; they had an average age of 12.3, and most of them had been diagnosed with cancer at the age of 9.6 on average. On average, they had been admitted three times, for a total of 15 days of hospitalization in the previous year. Their treatment-related medical expenses were usually paid by social insurance from the government or out of their own pocket.

Of the 80 parents, 78.8% were mothers of the children with cancer, with an average age of 40.5 years, and 43.8% were unemployed. Only 5.0% had primary education or below; 87.5% were married, and 31.3% had poor perceived economic status. About half did not have any religious beliefs.

#### Analysis among Children’s Symptom Burden, Parents’ Perceived Stress and Their Quality of Life According to Sample Characteristics

The absolute values of the z-statistic tests of skewness and kurtosis, parents’ perceived stress, quality of life, age, and educational level were <3.29, while those for children’s symptom burden, age, age of being diagnosed, number of admissions in the previous year, and number of hospitalization days in the previous year were >3.29, hence non-parametric tests were used for the variables from children. From Table 1, boys with cancer reported significantly higher symptom burden scores than their girl counterparts (*p* = 0.041), and the number of children’s hospitalization days in the previous year was associated positively with severity of symptom burden (*p* = 0.012). The mothers of the children reported significantly higher perceived stress scores than the fathers (*p* = 0.019), and the children’s ages were associated positively with parents’ perceived stress as well (*p* = 0.044). The parents’ quality of life was associated negatively with the children’s number of admissions in the previous year (*p* = 0.021) (Table 1).

As shown in Table 2, the cancer children reported a low level of symptom burden and the parents reported a low level of perceived stress and a moderate quality of life. MSAS scores were correlated positively with PSS-10 scores and negatively with WHOQoL scores, while PSS-10 scores were correlated negatively with WHOQoL scores; all three correlations were statistically significant (*p* < 0.05).

**Figure 1:**
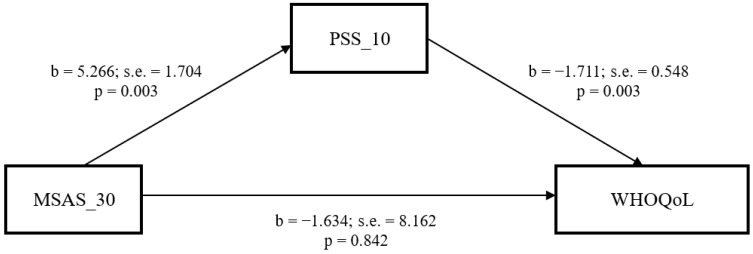
Mediating effect of parent’s stress on the relationship of children’s symptom burden with parent’s quality of life. Note: The model was controlled for child’s gender, relationship with child, no. of times of admission and no. of days of hospitalization.

Utilizing the PROCESS macro [22], a mediation model examined the direct and indirect effect of children’s symptom burden on parents’ quality of life through parents’ perceived stress (Figure 1) with the control of five covariates that were significantly associated with the study variables (relationship with the child, child’s gender, child’s age, number of admissions, and number of days of hospitalization). The bootstrapping results showed that parents’ perceived stress has a significant direct effect on their quality of life (B = −1.711, 95% CI = −2.807–0.615). The direct effect of children’s symptom burden on parents’ perceived stress was significant (B = 5.266, 95% CI = 1.858–8.674), but its effect on parents’ quality of life was not (B = −1.634, 95% CI = −17.958–14.690). The indirect effect of symptom burden on parents’ quality of life via parental stress was statistically significant (B = −9.010, 95% CI = −20.994–2.664).

## 5. Discussion

To our knowledge, this is the first study to examine how parental stress affects the relationship between Chinese children’s symptom burden and parents’ quality of life. Our results indicated that parental stress mediated this relationship. Specifically, the increased symptom burden of children with cancer might result in greater stress for parents, thereby negatively influencing parents’ quality of life. The findings of this study therefore provided an important insight into the potential mechanisms by which caring for a child with cancer adversely influenced parental quality of life. Understanding this mutual influence of dyadic interactions between parents and their children [10], as well as the factors contributing to parents’ quality of life could inform the development of the intervention. The evidence provided important insights in designing and implementing effective and sustainable interventions to support parents in managing their children’s health issues i.e., symptoms burden and thus also their own stress.

### 5.1. Priority Need to Improve Parents’ Quality of Life through Stress Reduction Program

Our findings indicated that child-reported symptom burden was highly associated with parental stress, which directly lowered parents’ quality of life. The result of this study was similar with the study done in western population that children’s symptom burden might play an important role in the development of parental distress [11]. Given the caregiving demands of their unwell children, parents were often confined to their homes due to caregiving (i.e., taking care of household activities, symptom management etc.) [24]. Parents might experience difficulty obtaining community resources and support from health professionals in managing their children’s health issues. Under such circumstances, parents might feel challenged, stress and lack of the confidence in taking care of the unwell child that causing the decrease of their quality of life [25]. Obviously, parents of children with cancer are more vulnerable to poor quality of life if they fail to monitor and mitigate the negative effects of their stress associated with their children’s symptom burden.

The literature suggested that increasing parents’ ability to manage their children’s health issues such as symptom burden may decrease the parental distress associated with caregiving activities in communities [25,26]. However, home visiting nursing services may not be sustainable because local health care system has been facing a serious nursing workforce shortage in recent years [27]. Besides, the paediatric community nursing services are affected or even suspended during the community outbreak of infectious disease, like the recent coronavirus disease pandemic. This situation shed light on the possibility of using telemedicine such as mobile application (App) program to support parents in stress reduction and managing their children’s health issues in order to improve their quality of lives. The delivery mode of App is considered to be suited to the existing local situation. Firstly, Hong Kong parents are familiar with the use of mobile App, since they often use it to search for health information [28]. Secondly, the App provides a platform to enhance nurse-parent interactivity and communication. Paediatric nurses can act as professional consultants in facilitating parents’ decision-making, not only in children’s health issues but also in stress management for themselves at home. It potentially maximizes the availability and accessibility of health service for both parents and children. The App intervention program will also contribute as a remote educational input to parents in relation to health assessment and management for both of the parents and their children with cancer. Therefore, a crucial initiative to develop a feasible community intervention for improving parental quality of life through stress reduction program is urgently needed [29].

### 5.2. Enhancing Nurse Competence in Supporting Parents in Symptom Management for Children with Cancer

According to our findings, parental stress was positively associated with the level of child-reported symptom burden, and negatively related to parents’ quality of life. Obviously, there is a service demand for paediatric community nurses to support parents in identifying and managing their children’s symptoms at home [30]. This proposed initiative simultaneously benefits parents’ quality of life and the alleviation of children’s symptom burden. Symptom management is considered the cornerstone of paediatric palliative care service [31]. It is defined as a core cancer care provided by health experts and focuses on the relief of symptoms with a goal of improving the quality of life of paediatric patients and their parents [32]. The result of this study showed that children with cancer have at least one symptom burden at some time, and that it concurrently induced stress in their parents and might cause children’ hospital admission. Given the potential of cancer and treatment-related symptoms, a comprehensive and accurate symptom assessment and management with appropriate triage are essential knowledge and skills for paediatric community nurses to ensure adequate symptom relief for children [33]. The paediatric community nurse is the key person providing direct care to the children living with cancer and their parents. They must be equipped with specialty nursing training in symptom management.

However, frontline clinicians were not usually comfortable addressing common symptoms such as pain seen in the paediatric population [31]. Owing to the limited standardized assessment tools and care plan provided by the health authority, some paediatric nurses reflected that they felt challenges not only in assessing but also in managing children’s symptoms [29]. They might perform symptom assessment, monitoring and outcome evaluation only based on their perceptions, best guesses, and experiences [34]. The misperceptions would let children’s suffering from symptom (i.e., pain, worry) persist. The majority of nurses in the study of Stacey et al. (2015) identified a need to improve their knowledge and skills in using symptom protocol [35]. As such, efforts to enhance nurses’ competences through post-registration training are required immediately in order to increase their abilities in symptom assessment, monitoring and management.

Of note is the fact that there is limited regular education provided in post-registration training programs with regard to symptom management for children living with cancer in the community. It is essential for frontline paediatric community nurses and health professionals to acquire knowledge in symptom assessment and develop a care plan, including pharmacological and non-pharmacological methods, for symptom monitoring and management. Ongoing professional training and support may help paediatric nurses to ensure their competences and consistent clinical practices in symptom management for paediatric oncology patients [29].

The evidence of this study provided important evidences for health educators and health professional policy makers to understand symptom burden commonly suffered by children living with cancer in communities meanwhile, negatively affecting their parents’ quality of life, which in turn can inform national and regional policy and health practice. This exploratory study was a crucial initial step in paving the way for the development of interventions to improve parental quality of life through stress reduction program. It also sheds light on post-registration nursing education and training on symptom management for health authorities to consider [36].

## 6. Limitations and Recommendations

The convenience sampling approach and small sample size used in this research study may limit the generalizability of the study results. Most participating parents were mothers (78.8%), so caution must be taken when generalizing results, since fathers’ interpretation of stress and their perceptions of their children’s quality of life might be different from those of mothers. Moreover, there is a variation of stress adjustment between western parents and Chinese parents [37,38]. Thus, the parental perceived stress level might be different between these two cultural groups, and the results may not be entirely applicable to other cultures. In addition, the internal consistency of PPS-10 was 0.603, which might reflect measurement faults among the participating parents in this study. Further psychometric testing of this assessment scale in samples of parents with children living with cancer would be warranted. Further research studies should investigate replication of the study to confirm the study findings across different regions and cultural backgrounds, with a larger sample size. This was a cross-sectional study that the findings were not able to allow for tracing potential time dynamics of parental stress level and their quality of life in different stages of tackling with the disease in a child. 

## 7. Conclusions

The results of this study enlarged the knowledge base about the mediating effect of parental stress on the associations between the symptom burden of children with cancer and their parents’ quality of life. Symptom burden is a common health concern in Chinese children with cancer. In this study, the children’s symptom burden was highly related to their parents’ stress, which in turn negatively influenced their parents’ quality of life. This evidence provides a clear direction for the development of interventions for improving parental quality of life through stress reduction program. Furthermore, in view of health professionals’ hesitations in symptom assessment and management for children living with cancer in the community, it was crucial to organize an educational training program to enhance the competences of paediatric nurses and other health professionals in symptom assessment, monitoring and management.

## Figures and Tables

**Table 1:** Children’s symptom burden, parent’s perceived stress and quality of life according to characteristics of parents and children (n = 80).

	MSAS	PSS-10	WHOQoL
	Mean ± SD/r_s_	Mean ± SD/r	Mean ± SD/r
Relationship with the child			
Father	0.25 ± 0.20	15.65 ± 4.40	117.41 ± 18.95
Mother	0.29 ± 0.30	18.33 ± 4.04	113.37 ± 18.52
*p*-value	0.967 ^a^	0.019 ^d^	0.429 ^d^
Parent’s age	0.173	0.098	0.058
*p*-value	0.124 ^b^	0.388 ^e^	0.610 ^e^
Parent’s employment status			
Full time	0.21 ± 0.19	17.07 ± 3.95	115.31 ± 18.51
Part time	0.30 ± 0.27	17.13 ± 4.44	121.56 ± 16.95
Unemployment	0.33 ± 0.34	18.63 ± 4.33	109.97 ± 18.62
*p*-value	0.442 ^c^	0.275 ^f^	0.108 ^f^
Parent’s education level	0.020	−0.007	0.179
*p*-value	0.862 ^b^	0.954 ^e^	0.113 ^e^
Parent’s marital status			
Single	0.38 ± 0.54	21.00 ± 4.24	95.00 ± 4.24
Married	0.26 ± 0.27	17.51 ± 4.22	114.51 ± 17.91
Divorced	0.53 ± 0.26	19.83 ± 4.49	121.50 ± 23.54
Widower/widowed	0.11 ± 0.09	17.00 ± 4.24	101.50 ± 30.41
*p*-value	0.114 ^c^	0.412 ^f^	0.262 ^f^
Family economic status			
Bad	0.34 ± 0.28	18.12 ± 3.50	108.00 ± 20.69
Good	0.24 ± 0.23	17.81 ± 4.38	117.00 ± 17.68
Very good	0.36 ± 0.53	16.14 ± 5.76	117.43 ± 11.93
*p*-value	0.354 ^c^	0.552 ^f^	0.129 ^f^
Have religion			
Yes	0.33 ± 0.31	18.48 ± 4.12	118.12 ± 18.05
No	0.26 ± 0.27	17.44 ± 4.28	112.45 ± 18.68
*p*-value	0.561 ^a^	0.310 ^d^	0.208 ^d^
Child’s gender			
Boy	0.34 ± 0.32	18.27 ± 4.81	113.41 ± 22.24
Girl	0.21 ± 0.22	17.14 ± 3.36	115.22 ± 12.98
*p*-value	0.041 ^a^	0.236 ^a^	0.651 ^a^
Child’s age	0.220	−0.226	0.159
*p*-value	0.050 ^b^	0.044 ^b^	0.158 ^b^
Child’s age of being diagnosed	0.107	−0.128	0.133
*p*-value	0.346 ^b^	0.259 ^b^	0.238 ^b^
Stage of disease			
Initial phase	0.27 ± 0.28	17.49 ± 4.21	114.79 ± 18.90
Recurrent	0.35 ± 0.28	19.33 ± 4.23	111.00 ± 16.93
*p*-value	0.220 ^a^	0.165 ^a^	0.517 ^a^
No. of admission in the past year	0.220	0.206	−0.257
*p*-value	0.050 ^b^	0.067 ^b^	0.021 ^b^
No. of hospitalization days in the past year	0.291	0.054	−0.135
*p*-value	0.012 ^b^	0.651 ^b^	0.256 ^b^
Deferment of study in the past year			
Yes	0.32 ± 0.32	17.80 ± 3.96	111.98 ± 18.24
No	0.23 ± 0.22	17.71 ± 4.64	117.26 ± 18.84
*p*-value	0.330 ^a^	0.919 ^a^	0.210 ^a^

^a^ Mann-Whitney U test; ^b^ Spearman correlation; ^c^ Kruskal-Wallis H test; ^d^ Independent *t* test; ^e^ Pearson correlation. ^f^ ANOVA.

**Table 2:** Children’s symptom burden and parents’ perceived stress and their quality of life (n = 80).

	Mean ± SD	Range	MSAS		PSS-10	
			r	*p*-Value	r	*p*-Value
MSAS	0.28 ± 0.28	0.00–1.52	-	-		
PSS-10	17.76 ± 4.32	4.00–26.00	0.376	0.001 ^a^		
WHOQoL	114.23 ± 18.56	78.00–164.00	−0.236	0.035 ^a^	−0.422	<0.001 ^b^

^a^ Spearman correlation. ^b^ Pearson correlation.

## Data Availability

The data presented in this study are available on request from the corresponding author. The data are not publicly available due to ethical issue.
